# Current diagnostic tools for coronaviruses–From laboratory diagnosis to POC diagnosis for COVID‐19

**DOI:** 10.1002/btm2.10177

**Published:** 2020-08-13

**Authors:** Yung‐Chih Wang, Yi‐Tzu Lee, Ting Yang, Jun‐Ren Sun, Ching‐Fen Shen, Chao‐Min Cheng

**Affiliations:** ^1^ Division of Infectious Diseases and Tropical Medicine, Department of Internal Medicine Tri‐Service General Hospital, National Defense Medical Center Taipei Taiwan; ^2^ Department of Emergency Medicine Taipei Veterans General Hospital Taipei Taiwan; ^3^ Faculty of Medicine, School of Medicine National Yang‐Ming University Taipei Taiwan; ^4^ Institute of Biomedical Engineering National Tsing Hua University Hsinchu Taiwan; ^5^ Institute of Preventive Medicine National Defense Medical Center Taipei Taiwan; ^6^ Department of Pediatrics National Cheng Kung University Hospital, College of Medicine, National Cheng Kung University Tainan Taiwan

**Keywords:** coronaviruses, COVID‐19, diagnostics, point‐of‐care

## Abstract

The Coronavirus‐2019 (COVID‐19) pandemic has put tremendous strain on healthcare systems worldwide. It is challenging for clinicians to differentiate COVID‐19 from other acute respiratory tract infections via clinical symptoms because those who are infected display a wide range of symptoms. An effective, point‐of‐care (POC) diagnostic tool could mitigate healthcare system strain, protect healthcare professionals, and support quarantine efforts. We believe that a POC tool can be developed that would be rapid, easy to use, and inexpensive. It could be used in the home, in resource‐limited areas, and even in clinical settings. In this article, we summarize the current state of COVID‐19 diagnostic methods and make a case for an all‐in‐one, highly sensitive POC assay that integrates antibody detection, protein detection, and serum cytokine detection to diagnose COVID‐19 infection. We believe this article will provide insights into the current state of diagnostics for COVID‐19, and promote additional research and tool development that could be exceptionally impactful.

## 1 INTRODUCTION

### 1.1 Facing the coronavirus threat head on

Coronaviruses are positive‐sense, single‐stranded RNA viruses belonging to the family *Coronaviridae*. They comprise four major genera, *Alphacoronavirus*, *Betacoronavirus*, *Deltacoronavirus*, and *Gammacoronavirus*, and some subgenera and species.^1^ Coronaviruses are so named because they have characteristic club‐shaped spikes projecting from their surface that resemble a solar corona when viewed with an electron microscope.^2^ Three coronaviruses belonging to the genus *Betacoronaviru*s, severe acute respiratory syndrome (SARS)‐CoV, Middle East respiratory syndrome (MERS)‐CoV, and SARS‐CoV‐2, have been responsible for outbreaks of severe human respiratory tract infection over the past 20 years.^3^, ^4^, ^5^, ^6^

First discovered in 2002, SARS‐CoV causes the deadly disease known as SARS.^5^, ^7^ This disease spread quickly from China to more than 30 countries, resulting in more than 8,000 cases and 700 deaths over 2 years.^5^, ^7^ Initially spread from camels to humans, MERS‐CoV causes the disease known as MERS. MERS was first observed in Saudi Arabia in 2012, and has since spread to several other countries.^5^, ^8^ SARS‐CoV‐2 is a new strain of coronavirus that was first identified in China in December of 2019.^9^ This virus, also named Coronavirus‐19 (COVID‐19) after the year in which it was discovered, has spread globally and resulted in the ongoing 2019–20 coronavirus pandemic.^10^

The diseases caused by these three coronaviruses display similar clinical symptoms including fever, dyspnea, malaise, myalgia, headache, and cough.^11^ These viruses are notorious for their rapid progression toward acute respiratory failure that can be fatal for a significant number of infected patients.^11^, ^12^ It is difficult for clinicians to distinguish them from each other based simply on their clinical presentations. The standard diagnostic tools for SARS, MERS, and COVID‐19 are summarized in Table 1. Real‐time reverse transcriptase–polymerase chain reaction (qRT‐PCR) is the gold standard approach for detecting the presence of viral RNA from respiratory specimens.^13^, ^14^, ^15^, ^16^ It can only be effectively used for disease diagnosis during the acute phase of infection, after which viral titers drop below the limit of detection. The advantages of qRT‐PCR include its high sensitivity and specificity for disease diagnosis. However, the accuracy of this approach relies on quality sample collection and transportation,^15^ and it can only be carried out by highly skilled technicians using expensive instrumentation. In addition, limitations in testing capacity and the availability of reagents render qRT‐PCR impractical for widespread application, a problem that is especially acute given the current pandemic.^14^

**TABLE 1:** Standard diagnostic tools for SARS, MERS, and COVID‐19

	Method	Sample	Advantages and disadvantages
SARS	1.Real‐time reverse transcriptase– Polymerase chain reaction (qRT‐PCR) for detection of SARS‐CoV 2.ELISAs^17^ and indirect IFA for detection of SARS IgG antibody	Nasopharyngeal swab; bronchial alveolar aspirates; blood	qRT‐PCR Pros: Sensitive, specific, and well‐developed technique Cons: Expensive, time‐consuming, well‐trained specialist needed, cannot detect recovered patients ELISA Pros: Well‐developed technique Cons: Requires large amount of sample specimen, limited in certain disease stages IFA Pros: Well‐developed technique Cons: Limited in certain disease stages, relatively subjective
MERS	1. qRT‐PCR for detection of MERS‐CoV 2. ELISAs for detection of antibodies	Bronchoalveolar lavage; sputum; tracheal aspirates; nasopharyngeal; oropharyngeal swabs; serum; stool	
COVID‐19	1. qRT‐PCR for detection of severe acute respiratory Syndromecoronavirus 2 (SARS‐CoV‐2)	Nasopharyngeal swabs; pharyngeal swabs; bronchoalveolar lavage fluid; sputum; feces; blood	

Abbreviations: COVID‐19, coronavius‐2019; ELISA, enzyme‐linked immunosorbent assay; IFA, immunofluorescent assay; MERS, Middle East respiratory syndrome; qRT‐pCR, real‐time reverse transcriptase–polymerase chain reaction; SARS, severe acute respiratory syndrome.

A serological assay is used to detect previous infection in people who have developed antibodies after exposure to the virus.^14^, ^16^, ^17^ It is a straightforward method for estimating the prevalence of a disease in a population by identifying individuals who have recovered from an infection and have established an immune response. This method is always employed during the convalescent phase of the disease but is not characteristically an element of early‐stage infection diagnosis because of the lag time associated with the adaptive immune system in the production of specific antibodies against the virus. Serological assays have played an important role in examining SARS epidemiology^18^ and other coronavirus outbreaks.^19^ Several serological assay approaches exist including enzyme‐linked immunosorbent assay (ELISA), immunofluorescent assay (IFA), and neutralization tests. The characteristic sensitivity and specificity of each approach varies.^14^, ^17^

Another approach for disease diagnosis is virus isolation in cell culture.^14^ To perform virus isolation, the active virus is isolated from a patient, replicated in live cells, and then harvested. Obtaining isolates for virus identification is important and very valuable, but such processes require specific cell lines and culture media, they are time‐ and labor‐intensive, and they require equipment and expertise.^14^ For these reasons, virus isolation is not suitable for clinical diagnosis.

### 1.2 Current laboratory diagnosis of COVID‐19

Since 2004, there have been no new, confirmed cases of SARS. MERS has a unique travel history and epidemiological association. Unlike SARS and MERS, COVID‐19 has spread to pandemic levels as designated on March 11, 2020 by the World Health Organization (WHO). It has spread rapidly and reached 188 countries, with more than 14,109,160 confirmed cases and 602,711 deaths as of July 18, 2020.^20^ Given the lack of effective medical treatments or vaccines, the only currently available disease management for COVID‐19 is to slow and reduce the spread of infection–this can be accomplished by early identification and patient isolation. On April 18, 2020, the US Food and Drug Administration (FDA) provided **Emergency Use Authorization (EUA**) (see Glossary) for several tests designed to detect SARS‐CoV‐2.^21^ Current diagnostic methods for detecting COVID‐19 are summarized in Table 2.

**TABLE 2:** Current diagnostic methods for COVID‐19

Sample	Method	Advantages and disadvantages	References
Blood	Nucleic acid detection (qRT‐PCR) or immunoassay for detection of IgG, IgM antibodies against SARS‐CoV‐2	Pros: Easy to operate; low infectious concern Cons: High false negative rate/high limit of detection (qRT‐PCR)	15, 72
Sputum	Nucleic acid detection (qRT‐PCR)	Pros: High sensitivity compared to pharyngeal swab Cons: High risk of infection for healthcare operators	15
Bronchoalveolar lavage	Nucleic acid detection (qRT‐PCR)	Pros: High detection rate/low limit of detection Cons: High risk of cross‐infection	15, 73
Nasopharyngeal swab	Nucleic acid detection (qRT‐PCR)	Pros: Most common sample specimen Cons: Possible concerns regarding false negative results; requires repeated detection	15
Pharyngeal swab	Nucleic acid detection (qRT‐PCR)	Pros: Easy to operate Cons: Less sensitive than nasopharyngeal swab and sputum; possible concerns regarding false negative results	15
Urine	Nucleic acid detection (qRT‐PCR)	Pros: Non‐invasive sample collection Cons: Limited data has been studied	15
Stool	Nucleic acid detection (qRT‐PCR)	Pros: Lower risk for healthcare infection compared to oropharyngeal swabs, non‐invasive Cons: Might be confined to later‐stage infection diagnosis	15, 74
Saliva	Nucleic acid detection (qRT‐PCR)	Pros: Lower risk for healthcare infection compared to oropharyngeal, pharyngeal swabs, and bronchoalveolar lavage Cons: Limited diagnostic tools available	30,31

Abbreviations: COVID‐19, coronavius‐2019; qRT‐pCR, real‐time reverse transcriptase–polymerase chain reaction.

SARS‐CoV‐2 has a single‐stranded RNA genome encoding 27 proteins, including an RNA‐dependent RNA polymerase (*RdRP*) and four structural proteins; (a) envelope protein (E); (b) matrix protein (M); (c) nucleocapsid protein (N); and, (d) spike surface glycoprotein (S).^22^ The receptor‐binding spike protein allows the virus to infect cells,^23^ and mediates receptor binding and membrane fusion, which determines host tropism and transmission capabilities.^23^ Compared to all previously described SARS‐related coronaviruses (SARSr‐CoVs), the S gene of SARS‐CoV‐2 is divergent with <75% nucleotide sequence similarity.^24^ The other three structural proteins are more conserved than the spike protein and essential for general coronavirus function.^22^

For detecting the presence of novel infectious diseases, the gold standard method has been the use of qRT‐PCR for the detection of viral RNA. The US Centers for Disease Control and Prevention (CDC) has developed the most widely used qRT‐PCR assay, which contains primer‐probe sets for two regions of viral nucleocapsid gene (N1 and N2), and for the human RNase *P* gene. However, the WHO primer‐probe sets target the *RdRP* and *E* genes. Until now, several different primers/probes sets have been developed globally for nucleic acid detection of SARS‐CoV‐2. Because SARS‐CoV‐2 belongs to a family of RNA viruses, mutation, and recombination are possible. It is, thus, difficult to effectively detect the virus using the same primers. The differences in primer selection may influence sensitivity and specificity for virus detection. Li et al. reviewed the list of published primers/probes and found that the conserved *E* gene is the target for the pan‐coronavirus assay, while *N* and *RdRP* genes are suitable for confirmatory assays.^25^ Implementation of qRT‐PCR is the most frequently used method for diagnosing COVID‐19 using respiratory samples,^26^ including upper respiratory samples (nasopharyngeal [NP] swabs, oropharyngeal [OP] swabs, NP washes, and nasal aspirates) and lower respiratory samples (sputum, bronchoalveolar lavage [BAL] fluid, and tracheal aspirates). An NP swab, rather than an OP swab, is recommended for early diagnosis or screening because of higher diagnostic yields, better patient tolerance, and reduced operator risk.^26^ Lower respiratory tract specimens yield the highest viral loads for the diagnosis of COVID‐19 and can be collected during or after the intubation procedure in patients with severe pneumonia and acute respiratory distress syndrome.^27^, ^28^ However, both BAL and tracheal aspirates are associated with a high risk for aerosol generation.^27^, ^28^ False negative results from respiratory samples could result from the variability in the detectable viral load, the number of days since the onset of illness, inadequate sampling techniques, low viral load in the area sampled, or mutations in the viral genome.^27^, ^28^ Aside from direct respiratory sampling, a rectal swab may be the preferred method in advanced COVID‐19 cases because high viral RNA of SARS‐CoV‐2 in fecal material has been noted in patients with COVID‐19 pneumonia late in their clinical course.^29^ Saliva has also been approved as a noninvasive specimen for detecting SARS‐CoV‐2.^30^, ^31^ The first saliva test for qualitative detection of SARS‐CoV‐2, ThermoFisher–Applied Biosystems TaqPath SARS‐CoV‐2 Assay (The Rutgers Clinical Genomics Laboratory), was approved (EUA) by the FDA in mid‐April, 2020.^32^ There are three issues associated with RT‐PCR for disease diagnosis: sophisticated laboratory equipment requirements, lengthy time demands, and the lack of any capacity for identifying asymptomatic patients who were infected with SARS‐CoV‐2 but have recovered.

Serological testing for SARS‐CoV‐2, an indirect detection of infection that measures the host response to infection, is facing increased demand because it is well suited for diagnosing COVID‐19 infection, even among asymptomatic or recovered patients. These tests can provide greater detail into the prevalence of a disease in a population, the role of asymptomatic infections, the basic reproduction number, and overall mortality. One potential challenge with developing accurate serological tests for SARS‐CoV‐2 includes cross‐reactivity with antibodies against other coronaviruses.^33^ Further, changes in viral load over the course of infection may make viral proteins difficult to detect. In contrast to viral load, antibodies generated in response to viral proteins may provide a larger window of time for indirectly detecting SARS‐CoV‐2. According to the FDA, IgM antibodies to CARS‐CoV‐2 are detectable in the blood just a few days after initial infection. However, IgM levels throughout the course of COVID‐19 infection are not well characterized. IgG becomes detectable 3 days after symptom onset or at least 7–10 days after infection.^34^ This limits the utility of serological detection for early‐stage diagnosis. To avoid the problem caused by changes in viral load over the course of infection that may make viral proteins difficult to detect, viral protein would be detected in the acute phase, with IgG/IgM detected in the convalescent phase. Further development of serological assays will be helpful for epidemiologic studies, ongoing surveillance, vaccine development, diagnosis/confirmation of late COVID‐19 cases, and for determining the immunity of healthcare workers as the outbreak progresses.

### 1.3 Opening the door for point‐of‐care diagnostics for COVID‐19

It takes approximately 4–6 hr for current qRT‐PCR techniques to provide diagnostic results for SARS‐CoV‐2 infection. A timely point‐of‐care (**POC) diagnostics** (see Glossary) method can shorten the time for diagnosis and enable physicians and medical staff to implement appropriate quarantine policies. This allows patients to receive immediate medical care and prevent further spread of the disease. Current POC diagnostics development can be divided into serological antigen or molecular detection methods (Table 3). Serological POC diagnostics are designed based on **lateral flow immunoassay (LFIA)** techniques (see Glossary). Several LFIA‐based POC tests have been approved by the FDA (EUA). One of these tests is the COVID‐19 IgM/IgG Rapid Test launched by Cellex Inc, which enables qualitative detection and differentiation of IgM and IgG antibodies to SARS‐CoV‐2 within 15–20 min.^35^ Another test is the DPP COVID‐19 IgM/IgG test developed by Chembio Diagnostics, which can provide results in just 15 min using a simple finger‐stick approach.^36^ The US FDA has authorized the first antigen test, which is designed based on the LFIA,^37^ and the second antigen test for rapid detection of the nucleocapsid protein antigen of SARS‐CoV‐2.^38^ These two assays provide timely diagnosis of those with active SARS‐CoV‐2 infection.

**TABLE 3:** Current available POC diagnostics for COVID‐19

Method	Sample	Design	Advantages and disadvantages
Serological antigen method	Blood, finger prick	Lateral flow immunoassay; colloidal gold immunochromatography	Pros: Rapid; low‐cost, and easy to use; suitable for disease surveillance Cons: Less sensitive in the early stage of disease
Molecular detection method	Throat swab, nasal swab, nasopharyngeal swab, saliva	PCR technique	Pros: High‐cost, rapid; easy to use Cons: Mostly are restricted in centralized clinical laboratories; difficulty in self‐sampling

Abbreviations: COVID‐19, coronavirus‐2019; POC, point‐of‐care.

Colloidal gold immunochromatography assay is a simple and fast detection tool widely applied in various fields for rapid diagnosis of disease.^39^, ^40^ It offers several advantages including speed, low cost, and ease of use, and it can be incorporated into a POC tool.^41^ Aytu Bioscience has developed a SARS‐CoV‐2 IgG/IgM Rapid Test using this technique. This test delivers results within 10 min, with a sensitivity and specificity of IgM of 89.2% and 100%, respectively.^42^ Although it has received Communate Europpene (CE) marking, it has not received approval by the US FDA.^42^ As of mid‐April of 2020, the US FDA has not reviewed any diagnostic tests based on colloidal gold immunochromatography technology.

Regarding molecular POC assays, all have been developed using PCR. Numerous assays have been developed by many companies and authorized for use by the US FDA (EUA). The Xpert® Xpress SARS‐CoV‐2 test (Cepheid, CA) is the first POC test approved by the US FDA (March 2020). This test can provide rapid detection of SARS‐CoV‐2 in just 45 min with less than a min of hands‐on time for sample preparation.^43^ One week later, the US FDA approved another molecular POC assay, the Accula SARS‐CoV‐2 Test (Mesa Biotech, CA).^44^ This assay utilizes PCR and lateral flow technologies for qualitative, visual detection of SARS‐CoV‐2 viral RNA in approximately 30 min. The Abbott ID NOW™ COVID‐19 test (Abbott, IL) is another prominent POC test.^45^ This test delivers high‐quality positive results in as little as 5 min, targeting the SARS‐CoV‐2 *RdRp* gene using isothermal nucleic acid amplification performed on Abbott's ID NOW™ platform—a lightweight box (6.6 pounds and the size of a small toaster). This is currently the fastest POC diagnostics tool for the detection of SARS‐CoV‐2.

Several companies in other countries have developed novel molecular POC assays for COVID‐19. One example is the VitaPCR™ COVID‐19 Assay produced by a Singapore company, Credo Diagnostics Biomedical.^46^ This assay can provide results in 20 min and has obtained a CE mark. Another well‐known test is the Vivalytic COVID‐19 test (Bosch, Germany), which delivers results in less than 2.5 hr using multiplex PCR and μArray‐detection to identify SARS‐CoV‐2.^47^

The abovementioned serological POC diagnostics are designed to detect antibodies in serum. An advantage of some serological POC diagnostics is that they can be performed on blood samples obtained by fingerstick rather than venipuncture. These methods are inexpensive, easy to use, and can provide results within approximately 20 min. A positive result indicates the development of an immune response to the infection, which allows these tests to be used for disease surveillance. Because antibodies most commonly become detectable 1–3 weeks after symptom onset, a positive antibody test does not imply acute infection. Molecular POC diagnostics are based on PCR, which is used to identify the presence of ORF1ab, spike, envelope, or nucleocapsid gene sequences associated with SARS‐CoV‐2 in throat swab, nasal swab, NP swab, or saliva samples. Compared to serological POC diagnostics, molecular POC diagnostics are more expensive and must be performed in a centralized clinical laboratory with suitable equipment. Most molecular POC diagnostics approaches require more than 20 min to produce results. A positive test result here indicates the presence of virus in the samples and the infectiousness state of the individual from which the sample was taken. Because positive test results vary depending on sample source and technique,^15^ negative results do not necessarily indicate absence of the virus.

The travel restrictions and social distancing policies recommended by the CDC have left many people around the world confined to their homes.^48^ Because of this, self‐administered testing can be effective. One diagnostic kit that merits particular note is the “Pixel” COVID‐19 At‐Home Test (LabCorp, NC) authorized by the US FDA on April 21, 2020.^49^ This kit allows people to collect a nasal swab sample at home and ship it back to the lab. This breakthrough innovation is an ideal diagnostic approach that is especially well suited for the COVID‐19 pandemic. As it does not require an in‐person visit to a medical professional, this collection method can protect frontline healthcare staff from exposure to symptomatic patients and conserve valuable time and personal protective equipment (PPE) resources. After LabCorp received a green light for their first at‐home kit,^49^ the US FDA approved the use of 10 additional at‐home collection kits for COVID‐19 as of June 30, 2020 (Table 4).^50^, ^51^, ^52^, ^53^, ^54^, ^55^, ^56^, ^57^, ^58^, ^59^ These kits allow people to self‐collect nasal swab or saliva samples outside of a healthcare setting and transported it to the manufacturer's testing laboratory. All of these tests employ PCR to detect the presence of SARS‐CoV‐2 RNA, and can provide results within 72 hr. While these kits are promising, their application is still limited. First, because all collected samples using these kits must be transported to the manufacturer's laboratory for analysis, they take longer to provide results compared to POC assays conducted in healthcare facilities. Second, all of these at‐home kits are designed to detect SARS‐CoV‐2 RNA during early‐stage infection, but they are not used to determine the presence of antibodies. Therefore, these tests cannot be used for surveillance and disease epidemiology purposes. Apart from PCR methods, the British‐based Iceni Diagnostics is developing a brand‐new approach that identifies SARS‐CoV‐2 via carbohydrate recognition using an artificial glycan receptor.^60^, ^61^ Another recent study conducted by Prof. Charles Y. Chiu at the University of California San Francisco in mid‐April of 2020 provides details regarding a CRISPR–Cas12‐based assay for detecting SARS‐CoV‐2.^62^ The authors of this study combined isothermal amplification using **reverse transcription loop‐mediated amplification (RT–LAMP)** (see Glossary) for RNA extracted with **CRISPR–Cas12** (see Glossary) DETECTR technology to create a novel POC test for detecting SARS‐CoV‐2 in clinical respiratory swab samples.^62^ Among 83 clinical samples tested, this test exhibited a positive predictive agreement and negative predictive agreement of 95% and 100%, respectively, relative to the CDC qRT–PCR assay for detection of the coronavirus in 30–40 min.^62^ Further confirmation experiments are needed before these approaches can be clinically applied. The US FDA authorized a CRISPR‐based COVID‐19 test (Sherlock CRISPR SARS‐CoV‐2 Kit) in May 2020. This is the first time the FDA allowed a CRISPR‐based tool to be used for patient testing.^63^ Prof. Seung Il Kim's group at Korea Basic Science Institute also proposed a field‐effect transistor‐based biosensing device with a specific antibody against SARS‐CoV‐2‐spiked protein for detecting SARS‐CoV‐2 in clinical NP swab specimens.^64^ This device detected target SARS‐CoV‐2 antigen protein with a limit of detection of 1 fg/ml which is low enough for practical use. In addition to this new device, the targeted spike protein used in this technique also aroused our interest.

**TABLE 4:** Currently available at‐home collecting kit for COVID‐19

Product	Manufacturer/institute	Sample type	Gene	Limit of detection	Performance evidence	Time to results	References
Pixel, COVID‐19 test home collection kit	Laboratory Corporation of America (LabCorp)	Nasal swab	Nucleocapsid (N) gene	6.25 copies/μl	A total of 30 participants enrolled in a self‐collection study. All positives (36/36) remained positive 72 hours post shipping. No false positives were detected (30/30)	Within 24 to 48 hr from receipt of the sample	48
Rutgers clinical genomics laboratory TaqPath SARS‐CoV‐2‐ Assay	Rutgers clinical genomics laboratory at RUCDR infinite biologics ‐ Rutgers university	Salvia	N gene, Spike (S) gene, *ORF1ab* gene	200 copies/ml	A study was conducted with 60 symptomatic patients showed 100% positive and negative agreement between the results obtained from testing of saliva and those obtained from nasopharyngeal and oropharyngeal swabs	Not available	49
Fulgent coronavirus disease (COVID‐19) RT‐PCR test	Fulgent therapeutics, LLC	Nasal swab	N gene	5 copies/μl	A total of 94 clinical specimens showed 100% concordance compared to a validated molecular assay	Within 24 to 48 hr from receipt of the sample	50
Everlywell COVID‐19 test home collection kit	Everlywell, Inc	Nasal swab	Collection kit only	N/A	N/A	N/A	51
P23 labs TaqPath SARS‐CoV‐2 assay	P23 labs, LLC	Saliva	N gene, S gene, *ORF1ab* gene	10 copies/μl	30 contrived positives and 40 contrived negative NP and saliva results produced the expected results	Within 72 hr	52
Quest self‐collection kit for COVID‐19	Quest diagnostics infectious disease, Inc	Nasal swab	N gene	136 copies/ml	There was 100% agreement for 30 positive samples and 30 negative samples	Within 24 to 48 hr from receipt of the sample	53
LetsGetChecked COVID‐19 home collection kit	PrivaPath diagnostics, Inc	Nasal swab	*ORF1ab* gene	83 copies/ml	There was 100% agreement for 69 positive and 109 negative remnant clinical nasopharyngeal specimens	2–5 days after lab receipt	54
Gravity diagnostics COVID‐19 assay	Gravity diagnostics, LLC	Nasal swab	N gene	2.4 copies/μl	There was 100% agreement for 30 positive samples and 30 negative samples	Within 48 hr of being received by laboratory	55
Phosphorus COVID‐19 RT‐qPCR test	Phosphorus diagnostics LLC	Saliva	N gene	5 copies/μl	There was 97.1% positive agreement f and 98.2% negative agreement	Available within 72 hr	56
KPMAS COVID‐19 test	Kaiser Permanente mid‐Atlantic states	Nasal swab	Envelope (E) gene, *ORF1ab* gene	25 copies/ml	There was 100% agreement for 50 positive samples and 100 negative samples	Within 24 to 72 hr of being received by laboratory	57
Kroger health COVID‐19 test home collection kit	Kroger	Nasal swab	Collection kit only	N/A	N/A	N/A	59

Abbreviations: COVID‐19, coronavirus‐2019; SARS, severe acute respiratory syndrome.

### 1.4 Perspective and future directions

**FIGURE 1:**
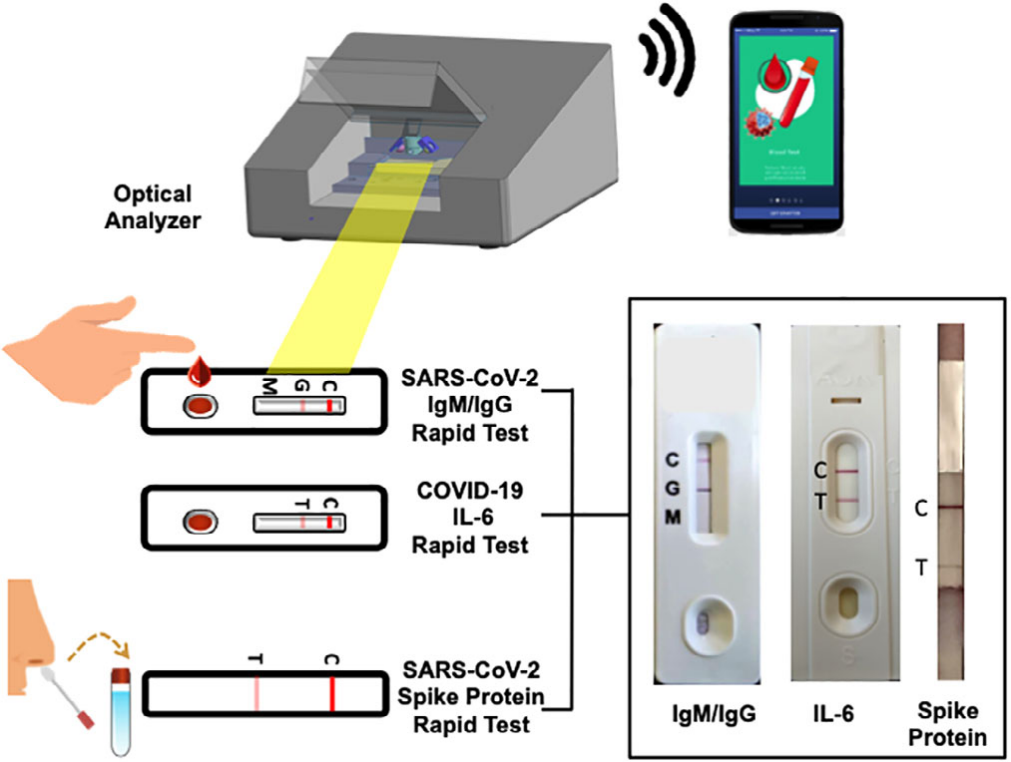
This proposed device is designed based on lateral flow immunoassay techniques. A nasal swab specimen is collected and used to detect SARS‐CoV‐2 spike protein. A finger stick blood sample can be obtained to detect SARS‐CoV‐2 IgM/IgG and IL‐6 levels. Test results are observed using an optical analyzer with good detection limits (photo credit: Hygeiatouch Inc. & Spectrochip Inc.). Key figure: Schematic of a point‐of‐care (POC) all‐in‐one device for diagnosis of coronavirus infectious disease‐2019 (COVID‐19). SARS, severe acute respiratory syndrome

As we mentioned above, molecular assays are more sensitive for use with patients in the acute infection phase, while serological tests are more suitable for use with patients with immunity and in the convalescent stage. Current POC diagnostics may not be suitable for all people suspected of having a COVID‐19 infection. Therefore, we propose a tool that would not only diagnose disease but differentiate disease severity. Previous studies have shown that the immunological profile of critically ill COVID‐19 patients demonstrated hyperactivation of the humoral immune pathway, including interleukin‐6 (IL‐6), which is a critical mediator for respiratory failure, shock, and multiorgan dysfunction.^65^, ^66^ Elevated serum IL‐6 levels predict imminent respiratory failure and correlate with acute respiratory distress syndrome in COVID‐19 patients.^65^, ^66^ Therefore, detection of IL‐6 titer in COVID‐19 patients with respiratory symptoms could be used to help physicians determine whether to prescribe ventilator usage. More importantly, cytokine release syndrome (CRS) occurs in a large number of patients with severe COVID‐19, which is also a significant cause of death.^67^, ^68^, ^69^ Consequently, cytokine storm treatment has become an important strategy for rescuing severely afflicted patients. Because IL‐6 is the key molecule of CRS, drugs blocking the IL‐6 signal transduction pathway could become a novel method for treating severely afflicted COVID‐19 patients. For this reason, IL‐6 receptor antagonists such as tocilizumab (Actemta™, Roche‐Genentch) and sarilumab (Kevzara™, Regeneron) may be useful as therapeutic agents to calm the inflammatory storm and reduce mortality.^67^, ^68^, ^69^ Prompt and serial detection of IL‐6 level in severely afflicted COVID‐19 patients could guide the use and timing of anti‐IL‐6 therapy. In accordance with our consideration, the US FDA has launched the first IL‐6 POC kit (The Elecsys IL‐6; Roche Diagnostics GmbH)^70^ for use in human serum and plasma specimens to provide early identification of severe inflammatory response and thus aid in determining the need for intubation and mechanical ventilation. We propose the development of an advanced POC diagnostics tool to detect SARS‐CoV‐2 IgM/IgG (with a detection limit of less than 1 ng/ml), SARS‐CoV‐2‐spike protein (with a detection limit of about 10^6^ plaque forming units per ml), and serum IL‐6 (with a detection limit of about 100 pg/ml) for the diagnosis of COVID‐19 based on LFIA and detection with an optical analyzer. This assay would detect both IgG and IgM using the finger‐stick method. A NP swab sample could be used to detect spike protein. The detection of both SARS‐CoV‐2 IgM/IgG (convalescent phase) and SARS‐CoV‐2‐spike protein (acute phase) could increase the diagnostic window, that is, from the early stage to 7 or 10 days of infection. In addition, determining IL‐6 level may facilitate early recognition of disease severity and prompt better patient management. The use of an optical reader device could also enhance detection threshold. Such a device could be conveniently integrated for use with a smartphone application, as mentioned in previous research,^71^ to provide instant and confidential diagnostic results (Figure 1). There are no differences in sampling method between the tools reviewed in this manuscript and the all‐in‐one POC diagnostic tool we propose. The proposed tool would be inexpensive, suitable for use at the site of triage or outside of hospitals and other healthcare facilities. It can be used frequently, at any and many timepoints, can be executed by staff or individuals with limited training, and requires little processing.

With the number of COVID‐19 patients needing acute and intensive care in healthcare systems surging, there is an urgent need for the development of a POC test for timely diagnosis and instant management. While there are several POC diagnostics tools available globally, some obstacles need to be overcome. First, most tools are limited to clinical laboratory use. These assays cannot be used at home or outside the healthcare system or laboratory setting. Although at‐home kits can collect samples outside of healthcare settings, it takes 24–72 hr to get results. Considering the number of people currently in at‐home quarantine situations, accessible POC diagnostic tools suitable for use outside of healthcare facilities, are highly desirable, but more ideal if they can provide more rapid, on‐site diagnosis. Also, because existing POC testing methods fail to detect the disease in both early and late stages, and they frequently provide false negative results, there is room for impactful improvement, and qRT‐PCR remains the diagnostic golden standard. There is a clear need for an easy to use, all‐in‐one POC diagnostics tool capable of detecting COVID‐19 infection during the whole course of the disease. The device we propose can overcome the weaknesses of current POC tests. We believe that this powerful, all‐in‐one diagnostics tool can shed light on the development and application of POC tests for detecting COVID‐19 infection and may positively impact disease management and alleviate healthcare system strain.

## GLOSSARY

### 1 Clustered regularly interspaced short palindromic repeat (CRISPR)

CRISPR‐Cas9 was adapted from a naturally occurring genome editing system in bacteria. The bacteria capture snippets of DNA from invading viruses and use them to create DNA segments known as CRISPR arrays that allow the bacteria to “remember” the viruses (or closely related ones). If the viruses attack again, the bacteria produce RNA segments from the CRISPR arrays to target the viruses' DNA, and then use Cas9 or a similar enzyme to cut the DNA apart, which disables the virus. The CRISPR‐Cas9 system works similarly in the laboratory. In this approach, a small piece of RNA is created with a short “guide” sequence that attaches to a specific target sequence of DNA in a genome. The RNA also binds to the Cas9 enzyme. As in bacteria, the modified RNA is used to recognize the DNA sequence, and the Cas9 enzyme cuts the DNA at the targeted location. Although Cas9 is the enzyme that is used most often, other enzymes (for example Cas12) can also be used. Once the DNA is cut, the cell's own DNA repair machinery add or delete pieces of genetic material, or make changes to the DNA by replacing an existing segment with a customized DNA sequence.

**Emergency Use Authorization (EUA):** a legal means for the Food and Drug Administration (FDA) in the United States to approve new drugs or new indications for previously approved drugs during a declared emergency.

**Lateral flow immunoassay (LFIA):** a type of solid‐phase immunoassay that combines the principles of thin‐layer chromatography and immune recognition reaction. In this assay, colored particles (colloidal gold, colloidal carbon, colloidal selenium, liposome, latex, quantum dot, etc.) can be selected as antibody labels to detect the presence of an analyte.

**Point‐of‐care (POC) diagnostics:** a test that is performed near or at the patient's side with the result leading to possible change in the care of the patient.

**Reverse transcription loop‐mediated isothermal amplification (RT‐LAMP):** a single‐tube technique for the amplification of DNA with high specificity, because of the use of 4 primers recognizing 6 distinct regions on the target base sequence, and rapidity due to the high amplification efficiency under isothermal conditions without the thermal cycler used in PCR. Each primer includes a target‐specific section of nucleotides and “tags” of contiguous nucleotides that are not complementary to the target sequences but allow the formation of loop structures. The detection of the amplified product is indirectly determined via the observation of turbidity produced from magnesium pyrophosphate, a by‐product of the amplification reaction. With the inclusion of reverse transcription (RT), the RT‐LAMP method allows for the detection of RNA.
